# Axial Compression Behavior of Ferrocement Geopolymer HSC Columns

**DOI:** 10.3390/polym13213789

**Published:** 2021-11-01

**Authors:** Taha Awadallah El-Sayed

**Affiliations:** Structural Engineering Department, Faculty of Engineering at Shoubra, Benha University, Cairo 11629, Egypt; taha.ibrahim@feng.bu.edu.eg

**Keywords:** axial behavior, geopolymer concrete (GC), ferrocement, finite element analysis (FEA)

## Abstract

Geopolymer concrete (GC) is a substantial sort that is created by utilizing metakaolin, ground granulated blast furnace slag (GGBS), silica fumes, fly ash, and other cementitious materials as binding ingredients. The current study concentrated on the structural behavior of the ferrocement geopolymer HSC-columns subjected to axial loading and produced using rice straw ash (RSA). The major goal of this research was to use the unique features of the ferrocement idea to manufacture members that function as columns bearing members. As they are more cost-effective and lower in weight, these designed elements can replace traditional RC members. The study also intended to reduce the cost of producing new parts by utilizing low-cost materials such as light weight expanded and welded wire meshes, polyethylene mesh (Tensar), and fiber glass mesh. For this purpose, an experimental plan was conducted and a finite element prototype with ANSYS2019-R1 was implemented. Nine geopolymer ferrocement columns of dimensions of 150 mm × 150 mm × 1600 mm with different volume-fraction and layers as well as a number of metallic and nonmetallic meshes were examined under axial compression loading until failure. The performance of the geopolymer columns was examined with consideration to the mid-span deflection, ultimate failure load, first crack load with various phases of loading, the cracking patterns, energy absorption and ductility index. Expanded or welded ferrocement geopolymer columns showed greater ultimate failure loads than the control column. Additionally, using expanded or welded columns had a considerable effect on ultimate failure loads, where the welded wire mesh exhibited almost 28.10% compared with the expanded wire mesh. Columns reinforced with one-layer of nonmetallic Tensar-mesh obtained a higher ultimate failure load than all tested columns without concrete cover spalling. The analytical and experimental results were in good agreement. The results displayed an accepted performance of the ferrocement geopolymer HSC-columns.

## 1. Introduction

The utilization of industrial by-products in the construction area is an important means of reducing construction costs and the safe removal of manufacturing waste [1,2,3,4,5]. In this sense, the straight use of alkaline fly ash and GGBS is recycled to manufacture geopolymer–cement for special construction concrete manufacturing [6,7]. The energy employed in geopolymer–cement production is much less than that used in OPC, which is directly affected by greenhouse gases [7]. In some adverse environmental conditions where OPCs are not very resistant, new and alternative concretes such as geopolymer–concrete need to be developed.

Most constructions use concrete hoists where OPC is used as the main binder. As a civil engineer, a lot is known regarding the environmental issues in the cement industry. The amount of carbon dioxide produced inside the atmosphere is equal to the manufacture of cement and it is similarly known that the energy required to produce cement is high, which consumes more fossil fuels [8,9]. When the cement is partially replaced in the existence of polymers and water at room temperature, the fly ash interacts with calcium hydroxide through the hydration procedure to produce a (C–S–H) gel. The application and development of large quantities of fly-ash in concrete has made it possible to replace cement up to 60% with a concrete mass [8].

Over the course of several years, wide-ranging research has been conducted to confirm the possibility of using GC as a building material [6,7,10,11,12,13,14].

The use of GC is gradually rising, particularly for chemical resistant structures in industries, and research is ongoing to expand the variety of applications. In fact, significant experimental work has been performed in Australia, the United States, and Spain. Several investigators have offered suitable starting materials for the production, stiffness, mix design, mechanical properties, and durability of GC [2,15,16]. The bearing capacity and stiffness of geopolymer concrete columns are influenced by the compressive strength of the material. Larger compressive strength geopolymer concrete columns provide a higher bearing capacity, stiffness, and ductility.

Ordinary concrete columns have a lower bearing capacity and rigidity than geopolymer concrete columns. In engineering applications, geopolymer concrete columns can meet the design requirements for structural columns with sufficient load capacity, stiffness, and ductility.

Mansur and Paramasivan [17] carried out a test study on ferrocement columns under centric and eccentric compressive loads. Test findings indicated that a ferrocement column could be used as a structural column. Kaushik et al. [18] executed a study on ferrocement RC columns and realized that the ferrocement enhanced the strength and ductility of the columns for centric and eccentric compressive loads. Similarly, various studies have been undertaken on different ferrocement structural elements under centric and eccentric compressive loads [19,20,21,22,23,24,25,26,27].

The major goal of this research was to use the unique features of the ferrocement idea to manufacture members that function as column bearing members. As they are more cost-effective and lower in weight, these designed elements can replace traditional RC members. The study also intended to reduce the cost of producing new parts by utilizing low-cost materials such as light weight expanded and welded wire meshes, polyethylene mesh (Tensar), and fiber glass mesh. Therefore, the main reason for this research was to examine the influence of the performance of RSA based geopolymer ferrocement HSC-columns under an axial compression load with various kinds and number of layers of metallic and nonmetallic meshes. For this purpose, an experimental plan was conducted and a finite element prototype with ANSYS2019-R1 was implemented. Nine geopolymer ferrocement columns with dimensions of 150 mm × 150 mm × 1600 mm were examined under axial compression loading until failure. The variables in this investigation were the mesh types and number of layers. The performance of the geopolymer columns was examined with consideration of the mid-span deflection, ultimate failure load, first crack load with various phases of loading, the cracking patterns, energy absorption, and ductility index.

## 2. Experimental Study

This experimental program was conducted in the Housing and Building National Research Center-Dokki-Egypt. A 5000-kN capacity test machine capable of testing columns up to 6 m in height was used to test the columns. The main aim was to find the ultimate deflection, ultimate load, and failure mode of the GC columns.

### 2.1. Materials

**Figure 1:**
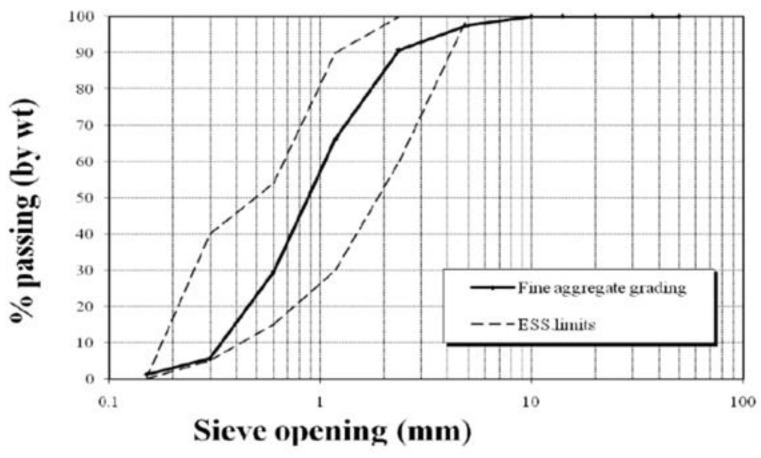
Fine aggregate grading curve.

**Figure 2:**
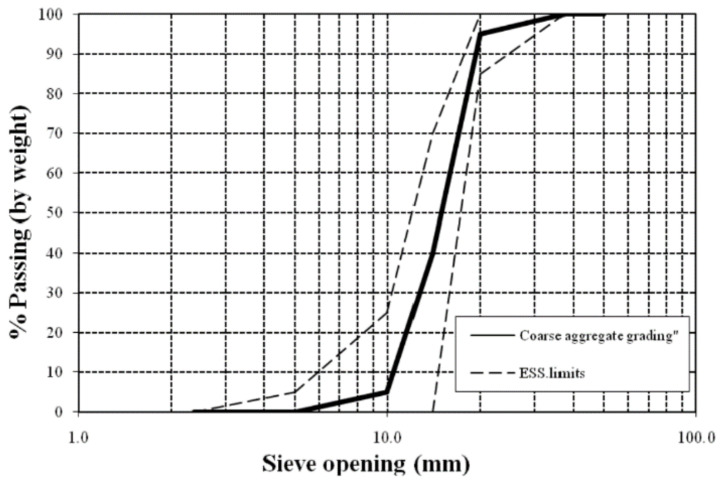
Coarse aggregate grading curve.

**Figure 3:**
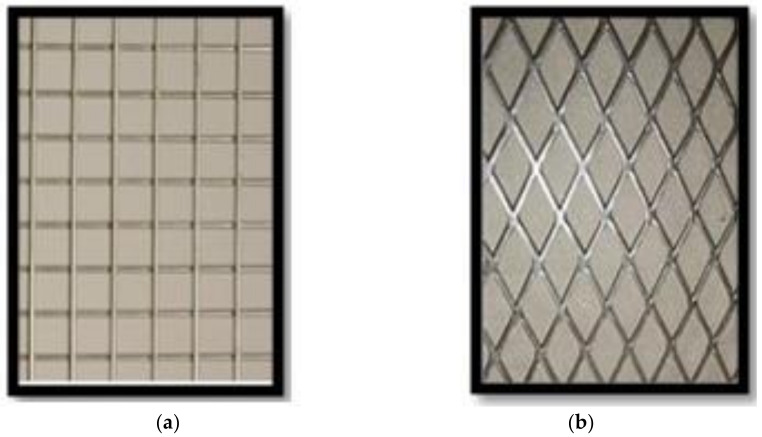
Types of meshes. (**a**) Welded wire-mesh, (**b**) Expanded wire-mesh [29].

**Figure 4:**
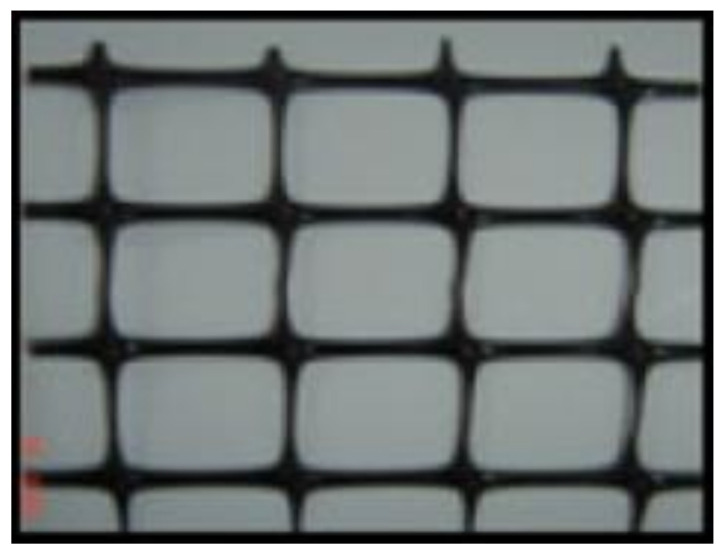
Polyethylene (Tensar) mesh.

**Figure 5:**
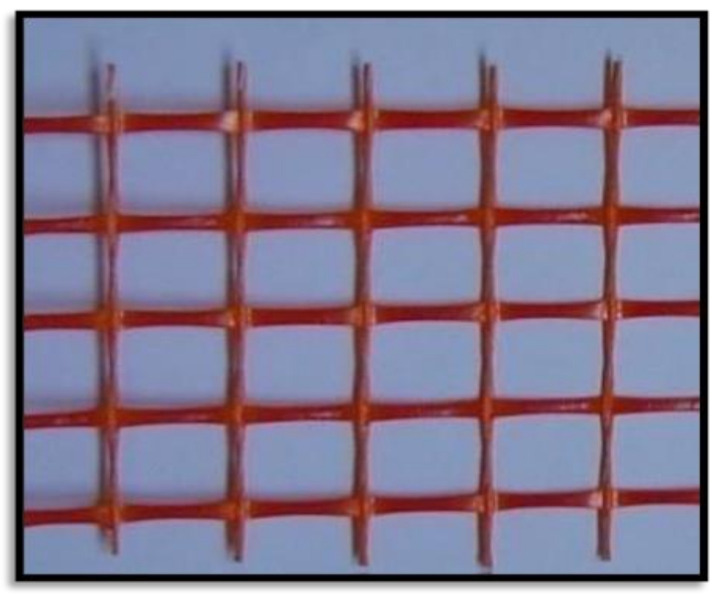
Fiber glass mesh.

1. Fine aggregate:
  Sand with 2.55 specific gravity and bulk density 1780 kg/m^3^. According to the Egypt Standard Specification (ESS) 203/2020 [28], a sieve analysis was performed. The results of the sieve analysis and the physical property test results are shown in Table 1 and Figure 1.
2. Coarse aggregate:
  Crushed aggregate with size 10 mm, 2.60 specific gravity and bulk density 1750 kg/m^3^. According to the Egypt Standard Specification (ESS) 203/2020 [28], sieve analysis was performed. The aggregate mechanical and physical properties are shown in Table 2, and the grading is shown in Figure 2.
3. Recycled Rice-Straw–Ash (RSA): RSA with a specific gravity 2.91 g/cm^2^, and specific surface area of 5200 cm^2^/g.
4. Water: used for mixing and curing.
5. Alkaline activator: sodium meta-silicate (Na_2_SiO_3_) and sodium hydroxide (NaOH).
6. Steel RFT: Two types of steel were used. Plain bars (24/35) with a 6 mm diameter, and deformed bars (42/60) with a 12 mm diameter.
7. Steel wire-meshes:
  (a) Welded and expanded wire-mesh:
    Figure 3 shows the types of ferrocement meshes used. Table 3 shows the mechanical properties of the welded and expanded steel wire-meshes.
  (b) Polyethylene (Tensar)-mesh:
    This mesh is made from the high density polyethylene, “Geogrid CE 121”, as shown in Figure 4, with an opening size of 6 mm × 8 mm, thickness of 3.3 mm, volume fraction of 2.04%, and weight of 725 gm/m^2^.
  (c) Fiber glass mesh:
    Gavazzi “V3-133-A” was used with an opening dimension of 12.5 mm × 11.5 mm. The cross-section dimension 1.66 mm × 0.66 mm (longitudinal direction) and 1.0 mm × 0.5 mm (transverse direction) as shown in Figure 5. The mesh has a volume fraction of 0.535% and weight of 123 gm/m^2^.

### 2.2. Design of Mix

Table 4 shows the mix design of the high strength concrete. The mix design was used to develop HSC at 28 days with a target strength of 60 MPa.

### 2.3. Column Sample Description

**Figure 6:**
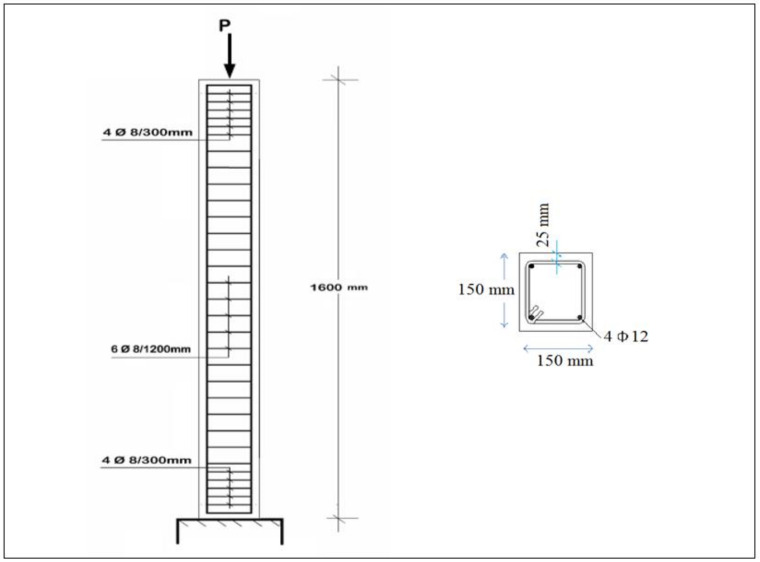
Concrete dimensions of columns and RFT details.

The experimental work was made to investigate the behavior, ultimate capacity, and crack-pattern of the geopolymer HSC columns. The experimental program consisted of (nine) geopolymer HSC columns with dimensions of 150 mm × 150 mm × 1600 mm reinforced with (4 φ 12) steel bars. Columns were tested axially using a compression machine of capacity 5000 kN. The concrete dimensions of the columns and details of RFT are presented in Figure 6. All tested columns are presented in Table 5. Additionally, Table 6 shows the reinforcement configurations for all tested columns.

### 2.4. Test Setup

**Figure 7:**
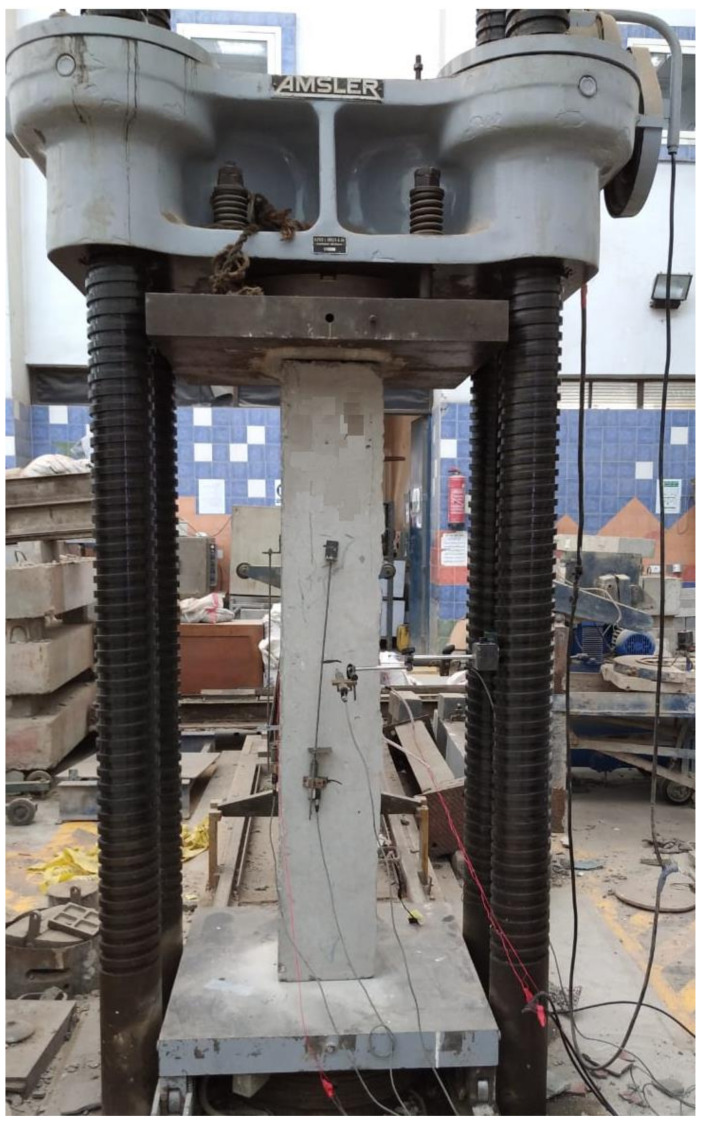
Test setup.

A compression test machine with a capacity of 5000 kN was utilized to test all column samples. Figure 7 shows a typical test setup for the columns. The deflection was measured using LVDT and all were tested until failure.

## 3. Discussion of Results

The behavior of the tested geopolymer HSC columns in terms of ultimate deflection, ultimate load, load–deflection relationship and failure mode, and cracking behavior are discussed as follows.

### 3.1. Ultimate Load

The ultimate loads for the tested columns are shown in Figure 11 and Table 7. The ultimate load of the control C1 was 738.70 kN. For group A, columns C1-A to C3-A, the ultimate loads extended between 771.80 kN and 955.60 kN. The improvement in the ultimate capacity was 4.30% to 22.70%. For group B, columns C4-B and C5-B, the ultimate loads extended between 813.70 kN and 821.70 kN. The improvement in the ultimate capacity was 9.20% and 19.90%. For group C, column C6-C, with an ultimate load of 1027.20 kN, there was a large considerable improvement of 28.10%. For group D, columns C7-D and C8-D, the ultimate loads were 793.40 kN and 841.30 kN, with an improvement of 7.20% and 12.20%, respectively.

According to the findings in Table 7, the use of fiber glass mesh is more efficient than other types of metallic and nonmetallic mesh reinforcements in increasing the ultimate capacity.

### 3.2. Ultimate Deflection

Figure 12 and Table 7 show the ultimate deflection for all tested columns. The deflection for the control C1 was 12.02 mm. For group A, the maximum deflection ranged from 12.72 mm to 14.50 mm for columns C1-A to C3-A, which were higher than that of the control C1. For group B, the maximum deflection at ultimate load was 15.45 mm and 16.96 mm for columns C4-B and C5-B respectively, which was also higher than that of the control C1. For group C, column C6-C, had an ultimate deflection of 13.55 mm. For group D, columns C7-D and C8-D, the ultimate deflections were 12.08 and 14.07 mm, respectively.

### 3.3. Load–Deflection Relationship

**Figure 8:**
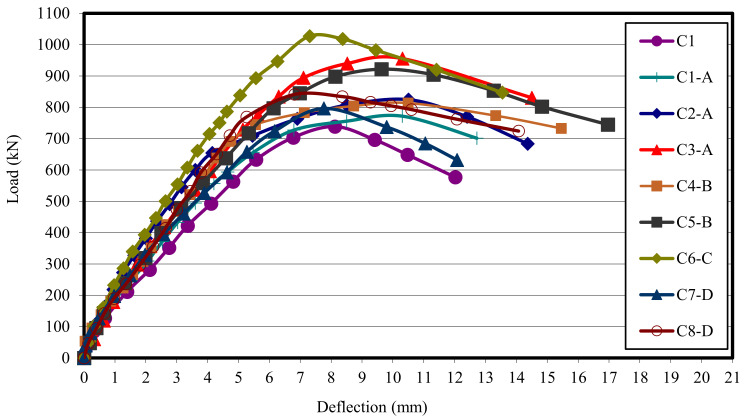
Comparison between the load deflection of all columns.

The relationship between the load and the deflection for the tested columns is presented in Figure 8. From this figure, it can clearly be seen that for all columns, the relationship between the load and deflection can be divided into three stages as follows:

1. Elastic behavior until the first cracking. The load–deflection relationship in this stage is linear. The slope of the load deflection curve in this stage varies with different types of test specimens. The end of this stage is marked by the deviation from linearity.
2. In the second stage, the load–deflection curve slope changed slowly as a result of the samples’ stiffness reduction due to the multiple cracking.
3. In the third stage, large plastic deformation occurred as the result of the yielding of the reinforcing bars and the large extension in the reinforcing mesh of the ferrocement columns. This stage is terminated by failure of the test columns.

**Figure 9:**
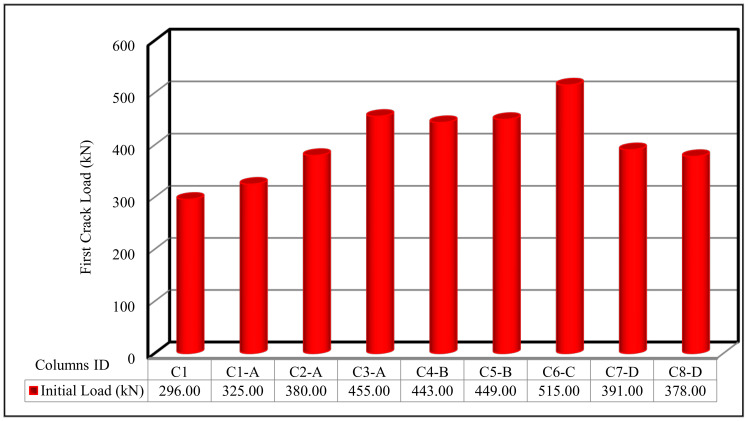
First crack load for the tested columns.

From Figure 9, it can be concluded that column C6-C, which was reinforced with one-layer of Tensar-mesh, had the highest first crack load and ultimate load, while the control column C1 had the lowest ultimate load carrying capacity.

The results of all test specimens are listed in Table 7. This table shows the obtained results for the first crack load, service load, deflection at ultimate load, ductility ratio, and energy absorption. First crack load, ultimate load, and first crack and ultimate load deflection were gained throughout testing, but the ductility ratio, service load, and energy absorption were calculated from the load–deflection curve for each column sample. The first crack-load was obtained at the point at which the curve of load deflection began to deviate from the linear relationship. Furthermore, service load can be computed from Equation (1).

(1) $$P_{ser}=\frac{\left(P_{ult}-1.4\times D.L.\right)}{1.6},D.L.=ownweightofcolumn.$$

**Figure 10:**
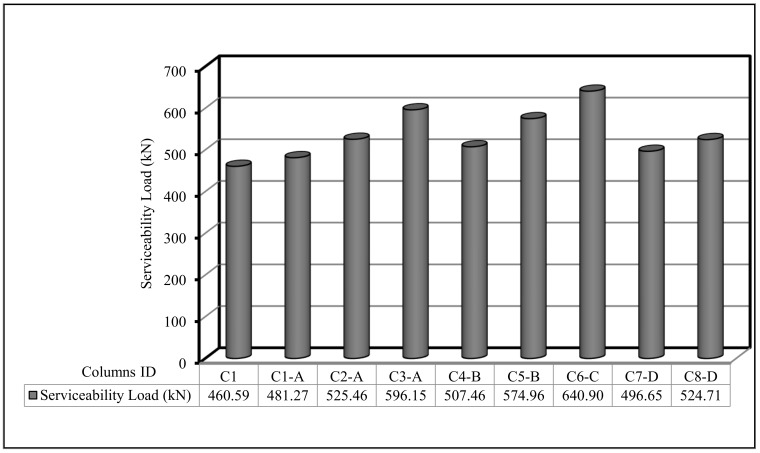
Serviceability load for the tested columns.

**Figure 11:**
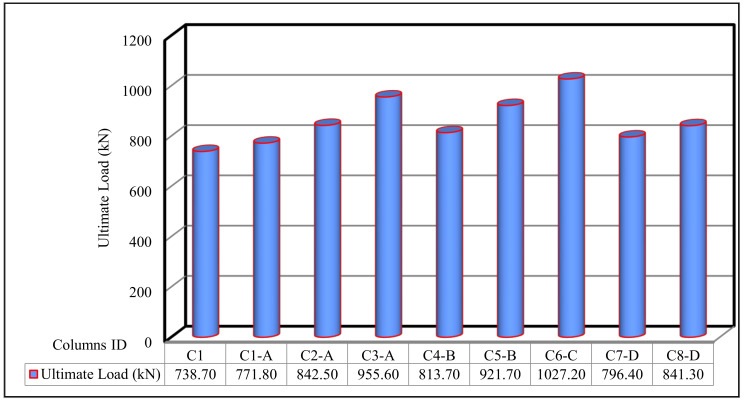
Ultimate load for the tested columns.

**Figure 12:**
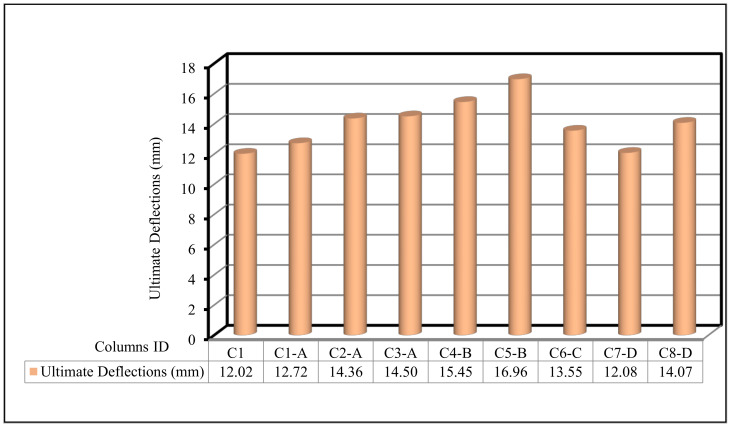
Ultimate deflection for the tested columns.

**Figure 13:**
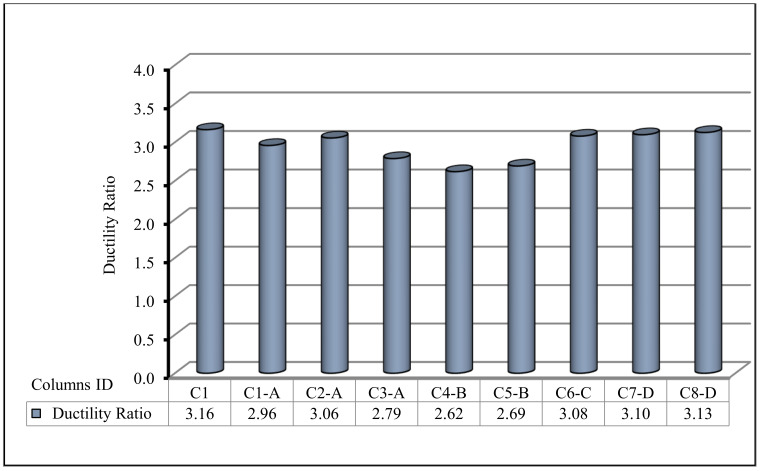
Ductility ratio for the tested columns.

**Figure 14:**
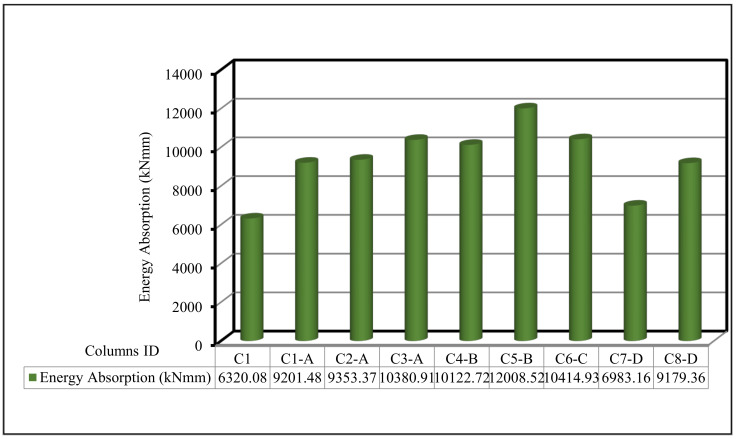
Energy absorption for the tested columns.

In the current section, the comparison between the behavior up to failure of the tested column as obtained from the experimental results is illustrated. The comparisons between all tested columns are shown in Figure 9, Figure 10, Figure 11, Figure 12, Figure 13 and Figure 14, which show the first crack load, serviceability load, ultimate load, deflection at ultimate load, ductility ratio, and energy absorption for all tested columns, respectively.

### 3.4. Energy Absorption

The ductility ratio was well-defined, similar to the proportion of the ultimate load deformation to the first crack load deformation, whereas the energy absorption was known as the area under the load deflection curve until collapse. Table 7 shows the ductility ratios and energy absorption values for all columns. Ongoing increase in the energy absorption as the volume fraction percentage increased, was noted. Column C8-D, with one layer of fiber glass mesh, had the highest ductility ratio when compared with the other types of meshes. Figure 14 shows the energy absorption comparison for all columns. The energy absorption for the control column C1 was 6320.08 kN·mm. For all other columns, the energy absorption was greater than the control C1. That is, it showed good enhancement with an enhancement percentage of 10% to 190%. Group D with the fiber glass mesh exhibited the smallest enhancement whereas Group C with the Tensar-mesh exhibited the highest enhancement. Column C5-B, which uses two layers of expanded wire mesh, had the highest energy absorption. It may be concluded that by improving the ductility ratio, these new composite materials improved the failure behavior.

Finally, the performance of columns was improved by employing these advanced composite materials. It may be said that it slowed the beginning of the first cracks, while also increasing the capacity of the service load. It also has high ultimate-loads, high durability, improved deformation, and improved energy absorption, all of which are advantageous in dynamic purposes.

### 3.5. Crack Pattern

**Figure 15:**
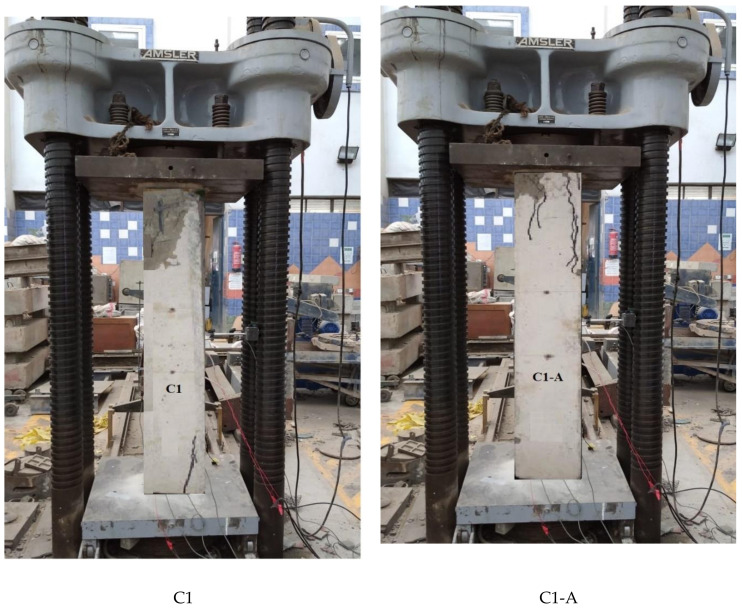

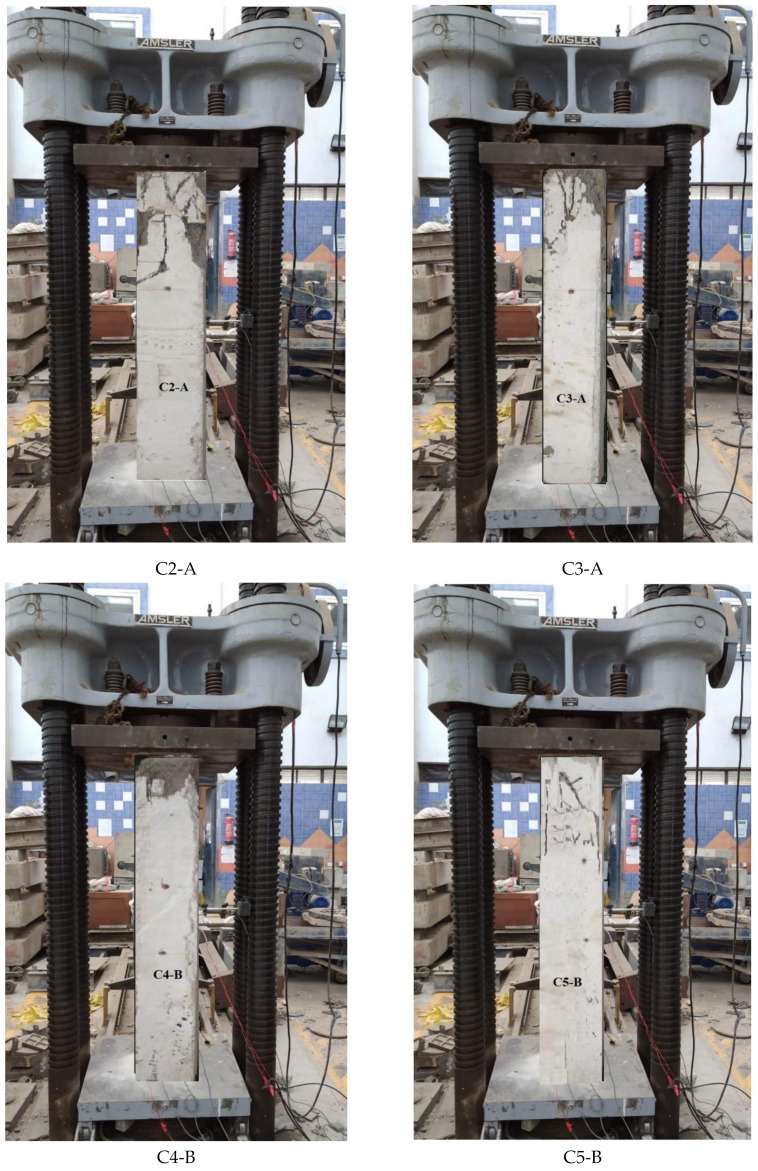

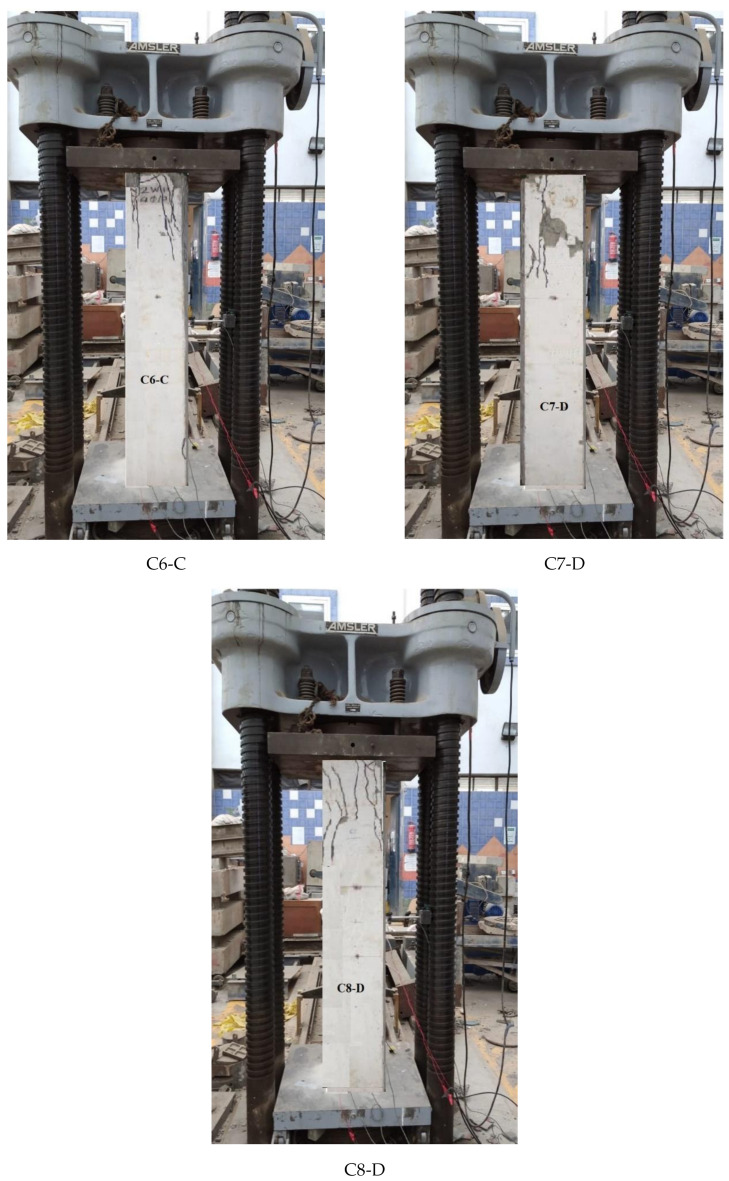
Crack patterns.

Near collapse, the control column C1 showed a compression mode of failure with a concrete cover of local crushing and spalling. For all other tested columns, near collapse after the ultimate load value decreased up to 60% to 40% of the ultimate load. All crack patterns of the tested columns are shown in Figure 15.

## 4. Analytical Analysis

Analytical analysis was conducted to validate the results of the experimental program. Table 8 showed the analytical results were obtained from the NLFEA software ANSYS2019-R1 [30] program.

### 4.1. Types of Elements

**Figure 16:**
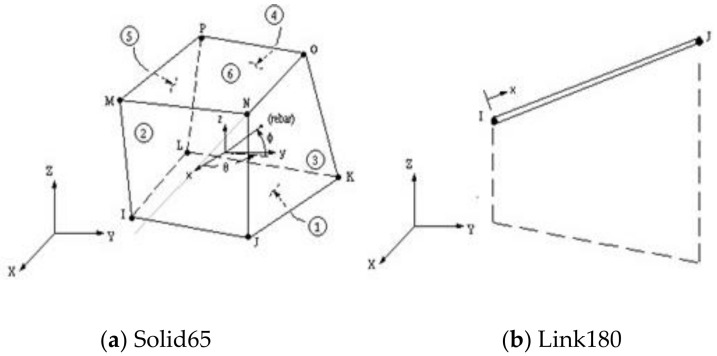
Geometry of element types.

For concrete, element Solid 65 (Figure 16a) was used to represent the concrete stress–strain curve, while element Link 180 3-D (Figure 16b) was used to represent the reinforcing bars and reinforcing stirrups. All ferrocement reinforcement was modeled by computing the volumetric ratio (reinforcing steel ratio to concrete) in the concrete element Solid 65. As ANSYS allows the user to enter three rebar materials in the concrete, each material corresponds to *x*, *y*, and *z*. The orientation angles denoted the reinforcement orientation in the smeared model. Therefore, ferrocement reinforcements were modeled as smeared layers with the volumetric ratio as indicated in Section 4.2.

### 4.2. Properties of Modeled Materials

This section shows the material properties for concrete, reinforcing steel bars, and ferrocement wire meshes:

- The material properties for concrete:
  1. Elastic modulus of elasticity (Ec = 4400√fcu = 24,100 N/mm^2^) [28].
  2. Poisson’s ratio (ν = 0.3) [28].
- The material properties for reinforcing steel bars:
  1. Elastic modulus of elasticity (*E_s_* = 200 kN/mm^2^) [28].
  2. Yield stress (*f_y_* = 400 N/mm^2^ & *f_yst_* = 240 N/mm^2^) [28].
  3. Poisson’s ratio (*ν* = 0.2) [28].
  4. Area of steel of φ 12 (As = 112 mm^2^)
  5. Area of steel of φ 8 (As = 50.3 mm^2^)
- The properties for welded mire mesh:
  1. Volumetric ratio of one layer = 0.0027
  2. Volumetric ratio of two layers = 0.0054
  3. Volumetric ratio of three layers = 0.0081
- The material properties for Expanded wire mesh:
  1. Volumetric ratio of one layer = 0.00753
  2. Volumetric ratio of two layers = 0.01510
- The material properties for Tensar mesh:
  1. Volumetric ratio of one layer = 0.02040
- The material properties for glass fiber mesh:
  1. Volumetric ratio of one layer = 0.00535
  2. Volumetric ratio of two layers = 0.01070

### 4.3. Specimens Modeling

**Figure 17:**
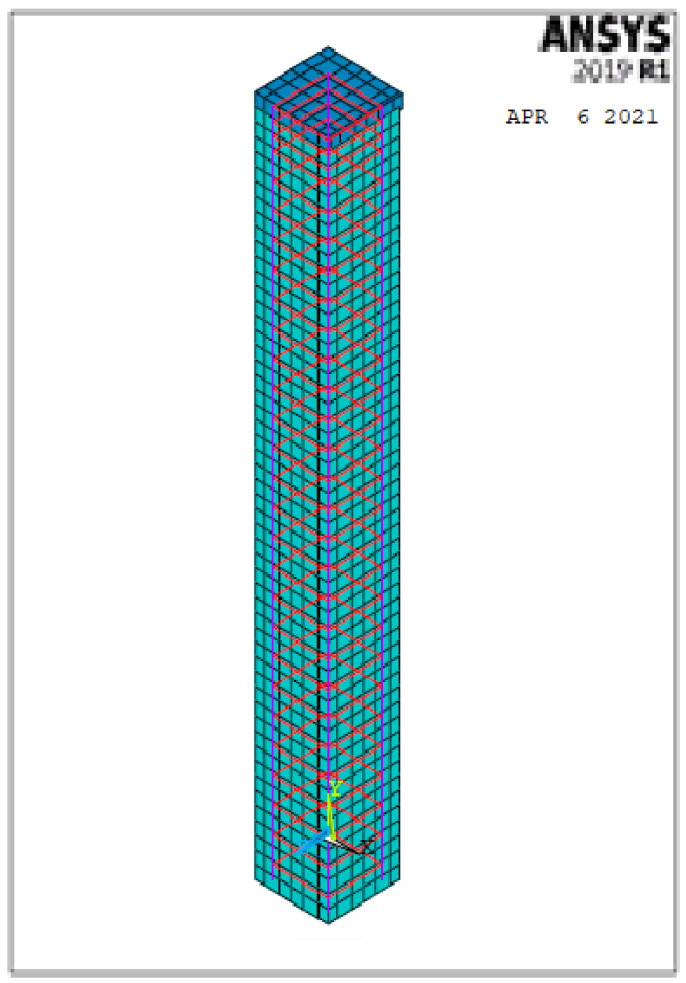
3D-modeling of the columns.

A finite nonlinear analysis was conducted to evaluate the behavior of geopolymer ferrocement HSC columns, as shown in Figure 17.

### 4.4. Analytical Results and Discussion

Table 8 shows the analytical results such as the first crack load, ultimate load, the deflection at first crack and ultimate load, ductility ratio, and energy absorption for the modeled columns.

#### 4.4.1. Ultimate Load

The ultimate loads for the modeled columns are shown in Table 8. The ultimate load of control C1 was 812.57 kN. For group A, columns C1-A to C3-A, the ultimate loads extended between 848.98 kN and 1051.16 kN. The improvement in the ultimate capacity was 4.40% to 22.80%. For group B, columns C4-B and C5-B, the ultimate loads extended between 995.07 kN and 1013.87 kN. The improvement in the ultimate capacity was 18.30% and 20.00%. For group C, column C6-C had an ultimate load of 1129.90 kN with a considerable improvement of 28.20%. For group D, columns C7-D and C8-D, the ultimate loads were 876.04 kN and 925.43 kN, with an improvement of 7.30% and 12.30%, respectively.

According to the findings in Table 8, the use of fiber glass mesh is more efficient than other types of metallic and nonmetallic mesh reinforcements in increasing the ultimate capacity. A good agreement was noted between the experimental and nonlinear approaches for all columns.

#### 4.4.2. Ultimate Deflection

Table 8 shows the ultimate deflection for all modeled columns. The deflection for the control column C1 was 8.12 mm. For group A, the maximum deflection ranged from 10.30 mm to 10.50 mm for columns C1-A to C3-A, respectively, which were higher than that of the control C1. For group B, the maximum deflection at ultimate load was 10.49 mm and 9.63 mm for columns C4-B and C5-B, respectively, which was also higher than that of the control C1. For group C, column C6-C, there was an ultimate deflection of 7.30 mm. For group D, columns C7-D and C8-D, the ultimate deflections were 7.76 and 6.82 mm, respectively. It can clearly be seen that good agreement was noted between the experimental and nonlinear approaches for all columns.

#### 4.4.3. Load–Deflection Relationship

The relationship between the load and deflection for the modeled columns are presented in Figures 21–29. From these figures, it can clearly be seen that good agreement was noted between the experimental and nonlinear approaches for all columns.

It is possible to conclude that the FE simulations produced accurate findings when compared to the experimental results. Furthermore, the analytical deflection findings outperformed the experimental results by a mean of 15%, as indicated.

#### 4.4.4. Energy Absorption

Table 8 shows the ductility ratios and energy absorption values for all modeled columns. The energy absorption for the control column C1 was 4104.83 kN·mm. For all other columns, the energy absorption was greater than the control C1. Group D with the fiber glass mesh exhibited the smallest enhancement whereas group C with the Tensar-mesh exhibited a considerable enhancement. It may be concluded that by improving the ductility ratio and the energy absorption, these new composite materials improved the failure behavior.

#### 4.4.5. Crack Pattern

**Figure 18:**
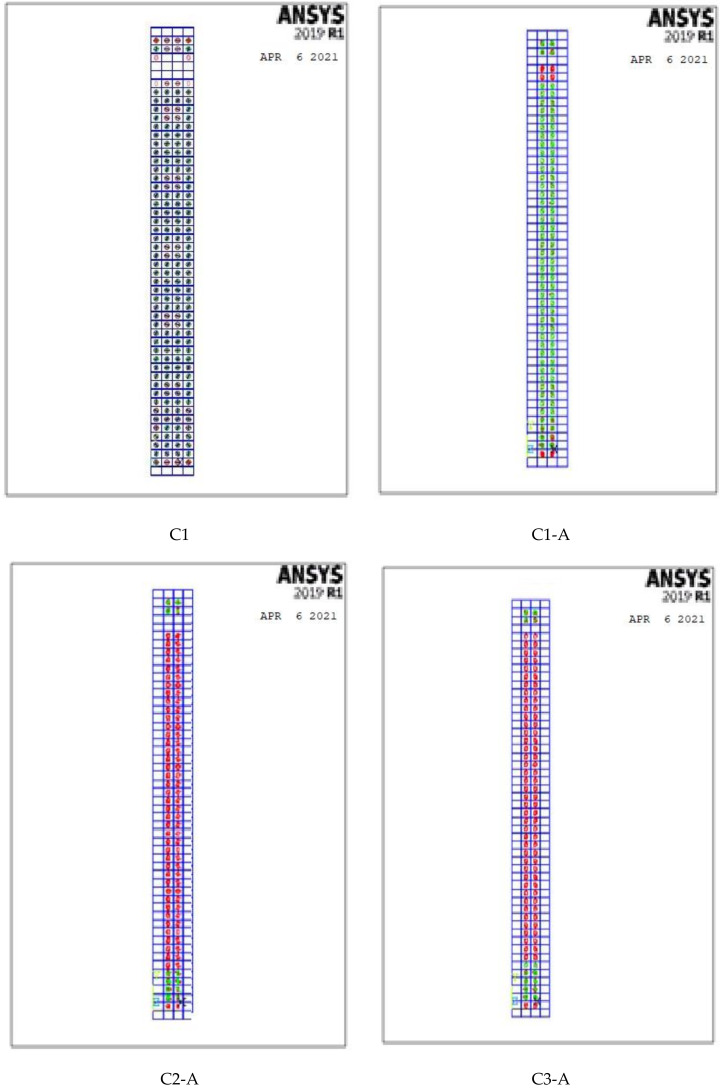

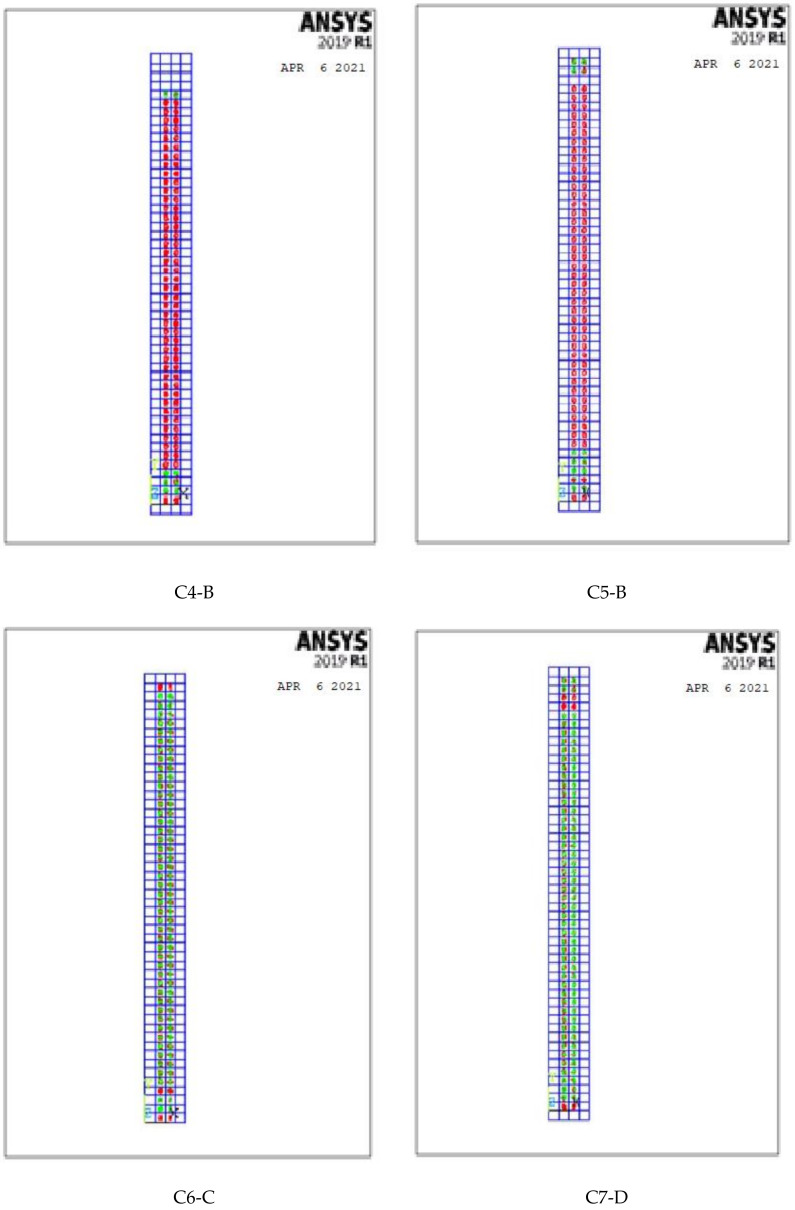

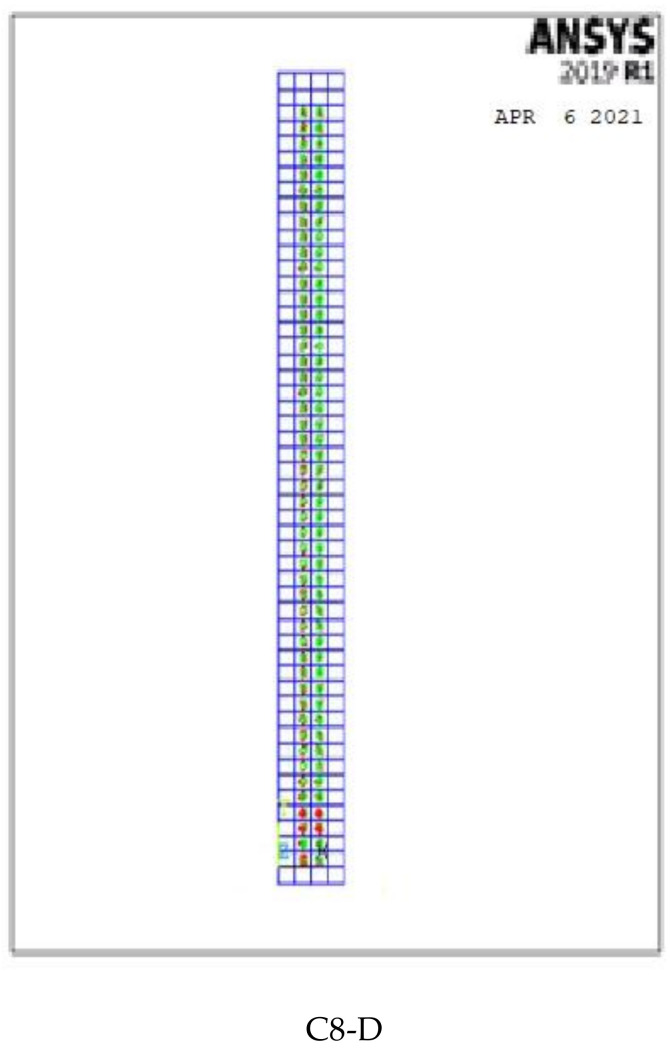
Crack patterns for the modeled columns.

The cracking started at an initial loading step in the molded column face near the column supports. This was due to the invisible micro-cracks in the experimental study. The cracking load was fairly lower than the experimental one. This might be acceptable as the FE analysis characterized the stage of micro-cracks. Furthermore, the wire mesh composite materials may have hidden the micro-cracks initiated in the experimental test. Conversely, the patterns of cracks at every load step showed that the crack propagation for all columns was somewhat changed compared to the experimental one due to the accuracy of the FE Ansys program in obtaining the micro-cracks. All crack patterns of the molded columns are shown in Figure 18.

## 5. Comparisons between Analytical and Experimental Results

Comparison between analytical and experimental results confirmed an acceptable agreement in representing the geopolymer ferrocement HSC columns’ performance in terms of first crack and ultimate deflection, first crack load and ultimate load, and crack pattern.

### 5.1. Ultimate Failure Load

**Figure 19:**
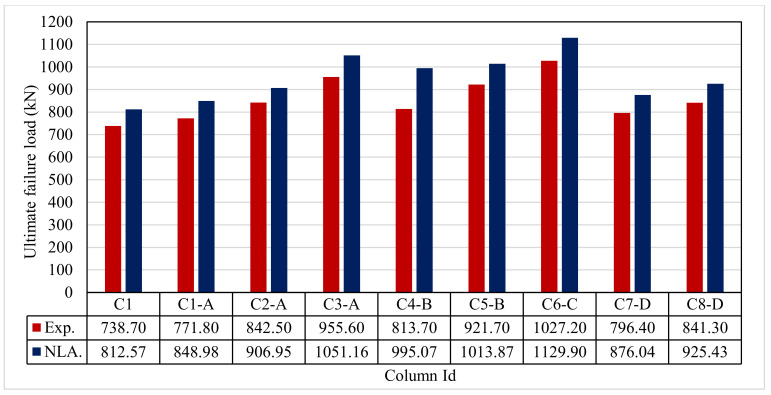
Comparison between Exp. and NLA ultimate loads.

Figure 19 and Table 9 show the comparison between the ultimate experimental and analytical load. There was fair agreement between the experimental and analytical ultimate loads. It is possible to conclude that the FE simulations produce accurate findings when compared to the experimental results. Furthermore, the analytical ultimate load results outperformed the experimental results by a mean of 11%.

### 5.2. Ultimate Deflection

**Figure 20:**
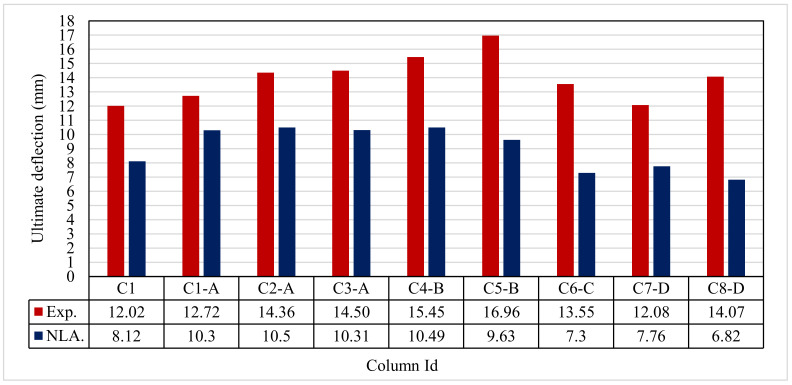
Comparison between Exp. and NLA ultimate deflections.

**Figure 21:**
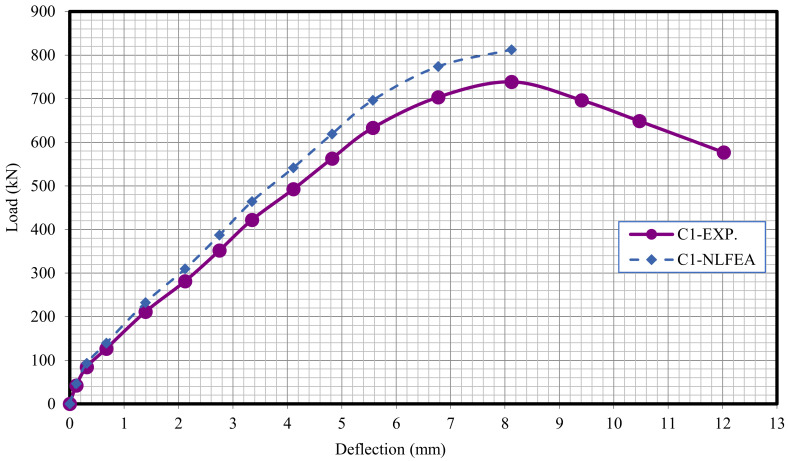
Load deflection of Column C1.

**Figure 22:**
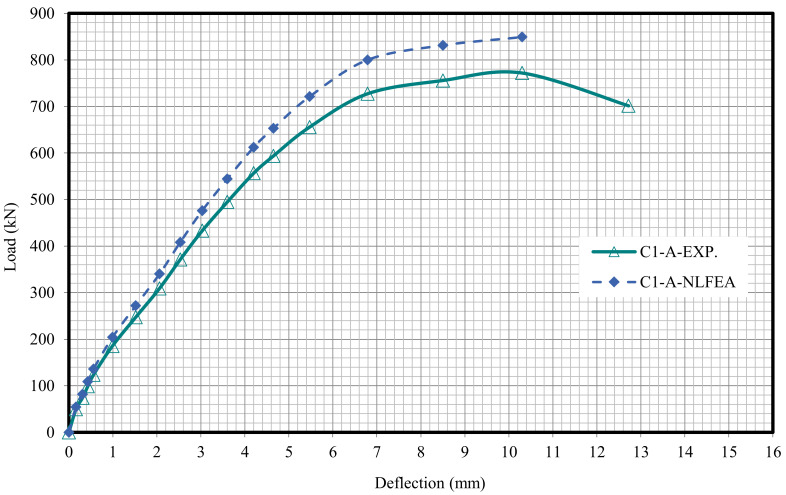
Load deflection of Column C1-A.

**Figure 23:**
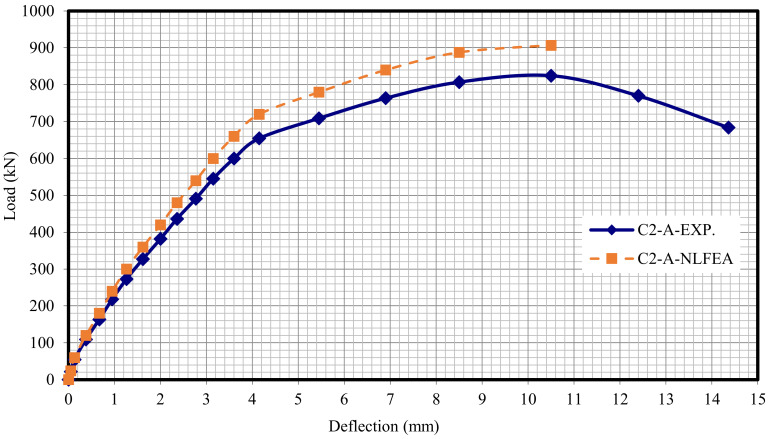
Load deflection of Column C2-A.

**Figure 24:**
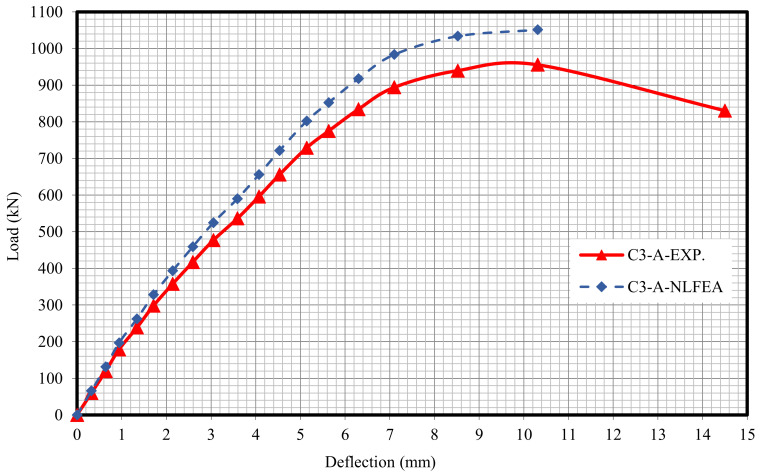
Load deflection of Column C3-A.

**Figure 25:**
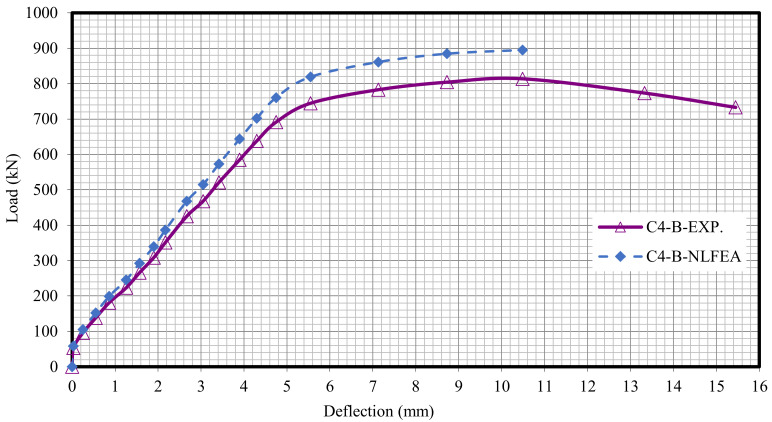
Load deflection of Column C4-B.

**Figure 26:**
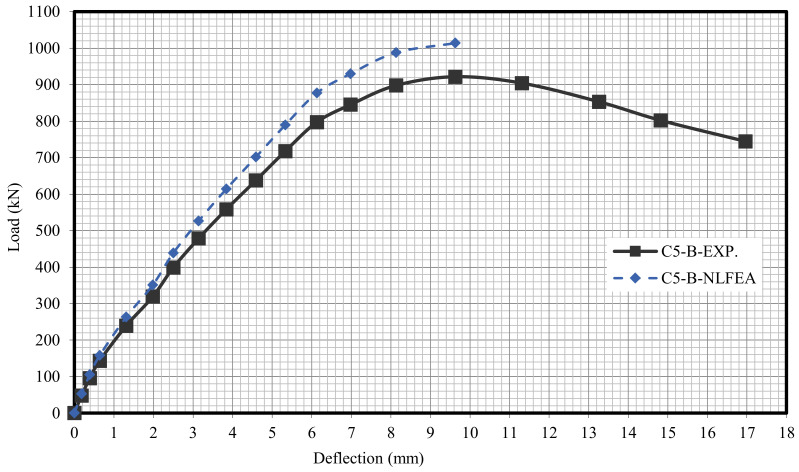
Load deflection of Column C5-B.

**Figure 27:**
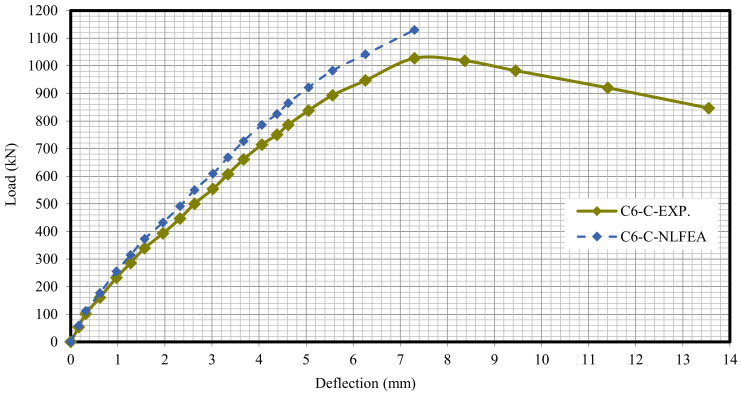
Load deflection of Column C6-C.

**Figure 28:**
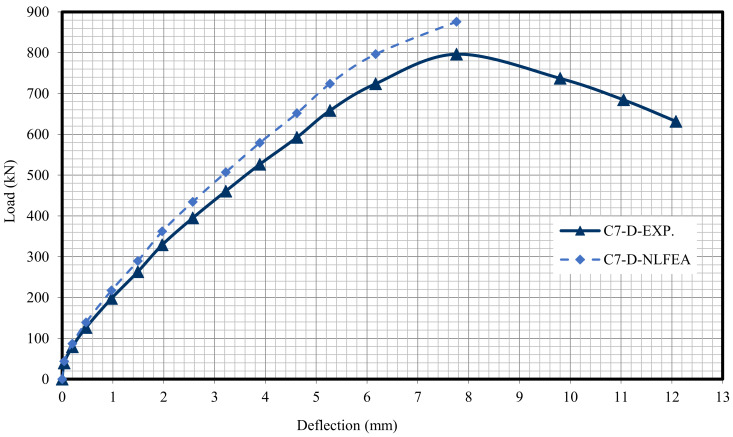
Load deflection of Column C7-D.

**Figure 29:**
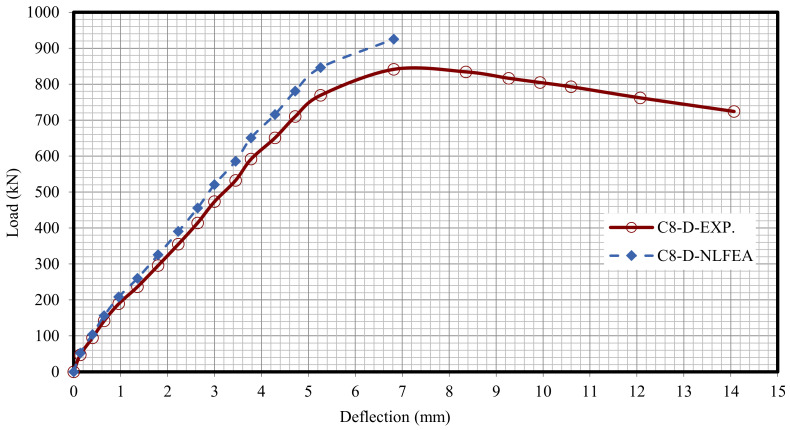
Load deflection of Column C8-D.

Figure 20 and Table 9 show the comparison between the ultimate experimental analytical deflections. The load–defection curves as shown in Figure 21, Figure 22, Figure 23, Figure 24, Figure 25, Figure 26, Figure 27, Figure 28 and Figure 29 for the experimental and modeled columns showed good agreement with respect to the control column deflection. Furthermore, the analytical ultimate defection results outperformed the experimental results by a mean of 15%.

### 5.3. Cracking Patterns

**Figure 30:**
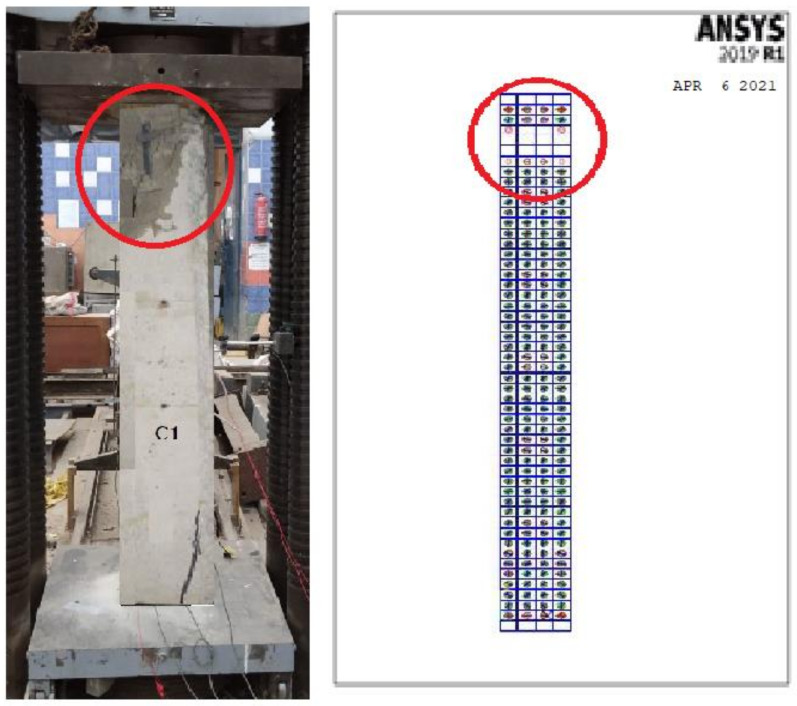
Crack spread for the control specimen.

**Figure 31:**
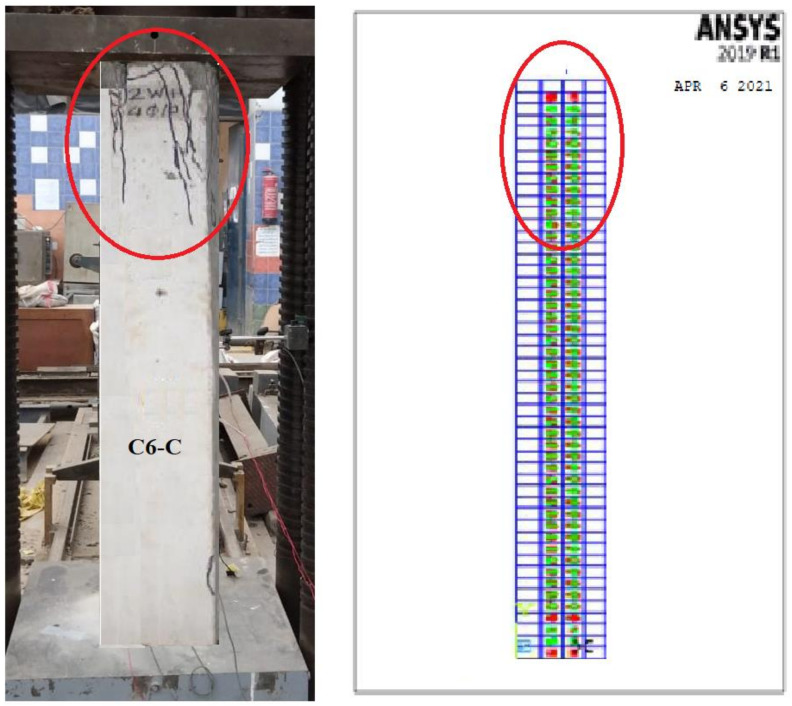
Cracks spread for the C6-C specimen.

Figure 30 and Figure 31 show the comparison between the crack pattern for the experimental and modeled column samples. The micro-cracking stage, which occurs before observable cracking, is represented by the NLA forecasts. The cracking patterns at each load increment, on the other hand, demonstrated that the crack propagation for all columns differed somewhat from the experimental fracture pattern. This is due to the nonlinear finite element program’s precision in determining micro- and large cracks as well as the impact of the reinforcing technique on cracking patterns, as shown in Table 9.

## 6. Conclusions

Based on the experimental and analytical results, the following conclusions can be drawn:

1. Because of the lighter and easier handling of wire meshes compared with steel reinforcement, all wire meshes offer several improvements, especially for structures with complex shapes.
2. Increasing the volume fraction of the wire mesh reinforcement increased the initial cracks, ultimate loads, energy absorption, and ductility index.
3. Ferrocement geopolymer columns achieved higher ultimate load, ductility, and energy absorption compared to the steel reinforced concrete control column.
4. Cracks with greater number and narrower widths were observed for those ferrocement geopolymer columns compared with the steel geopolymer columns.
5. Expanded or welded ferrocement geopolymer columns showed greater ultimate failure loads than the control column. Additionally, using expanded or welded columns had a considerable effect on the ultimate failure loads, where welded wire mesh exhibited almost 28.10% compared with expanded wire mesh.
6. Column reinforced with one-layer of nonmetallic Tensar-mesh obtained the highest ultimate failure load out of all the tested columns without concrete cover spalling. Consequently, increasing the volume fraction had the main result of postponing the incidence of crack development with higher corrosion protection and high loading carrying capacity than columns reinforced with metallic reinforcement.
7. Column reinforced with one layer of fiber glass mesh obtained the smallest ultimate failure load compared with the control column.
8. The analytical procedures for first crack and ultimate load computations obtained good prediction for these loads and the column failure modes. Consequently, there were improved strength, deformation characteristics, and cracking behavior with great savings of reinforcement.
9. The comparison of the crack patterns obtained by the FE and experimental models led to identical crack propagation for the two approaches up to failure. The inclination of the failure surfaces and the concentration of cracks of all columns were the same in both patterns.
10. The established ferrocement geopolymer columns could be successfully used as an alternative to the traditional RC columns, which could be of true merit in both developed and developing countries aside from its anticipated economic and environmental merits.

## Figures and Tables

**Table 1:** Physical properties of the sand.

Property	Results	ESS Acceptance Limits
Specific gravity (kg/m^3^)	2.55	-
Bulk density (kg/m^3^)	1780	-
Materials finer than no. 200, sieve (0.074 mm)%	1.4	Less than 4%

**Table 2:** The physical and mechanical properties of the coarse aggregate.

Property	Results	ESS Acceptance Limits
Specific gravity (kg/m^3^)	2.2.60	-
Unit weight (kg/m^3^)	1750	-
Absorption Percentage	1.46%	No more than 2.5%

**Table 3:** Mechanical properties of the welded and expanded wire-meshes [29].

Welded Wire Mesh		Expanded Wire Mesh	
Dimensions size	12.5 × 12.5 mm	Dimensions size	16.5 × 31 mm
Weight	600 gm/m^2^	Weight	1660 gm/m^2^
Thickness	0.7 mm	Wire Diameter	1.25 mm
Young’s Modulus	17000 N/mm^2^	Young’s Modulus	12000 N/mm^2^
Yield Stress	400 N/mm^2^	Yield Stress	250 N/mm^2^
Yield Strain	1.17 × 10^−3^	Yield Strain	9.7 × 10^−3^
Ultimate Strength	600 N/mm^2^	Ultimate Strength	380 N/mm^2^
Ultimate Strain	58.5 × 10^−3^	Ultimate Strain	59.2 × 10^−3^

**Table 4:** Mix design of HSC.

Item	RSA	Coarse Aggregate	Fine Aggregate	NaOH	Na_2_SiO_3_	Water
	(kg/m^3^)	(kg/m^3^)	(kg/m^3^)	(kg/m^3^)	(kg/m^3^)	(kg/m^3^)
Per m^3^ of concrete	400	1150	650	50	150	47

**Table 5:** Description of the studied columns.

Series	Sample	Sample Description	Volume of Fraction	RFT.	Stirrups
	ID				
Control	C1	Control	---------	4 φ 12	6 φ 8/’
Group A: Welded wire-mesh	C1-A	1-layer welded	0.00270	4 φ 12	------
	C2-A	2-layers welded	0.00540	4 φ 12	------
	C3-A	3-layers welded	0.00810	4 φ12	------
Group B: Expanded wire-mesh	C4-B	1-layer expanded	0.00753	4 φ 12	------
	C5-B	2-layers expanded	0.01510	4 φ 12	------
Group C: Tensar-mesh	C6-C	1-layer Tensar	0.02040	4 φ 12	------
Group D: Fiber glass-mesh	C7-D	1-layer fiber glass	0.00535	4 φ 12	------
	C8-D	2-layers fiber glass	0.01070	4 φ 12	------

**Table 6:** Reinforcement configurations of the tested columns.

Sample	Tested Columns Reinforcement Configurations
ID	
C1	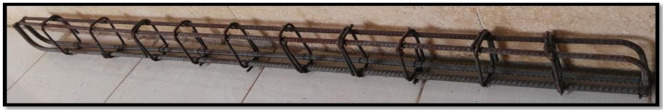
C1-A	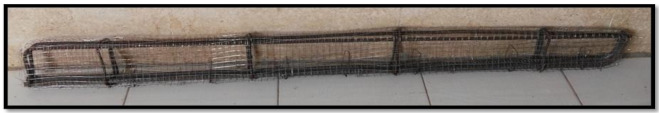
C2-A	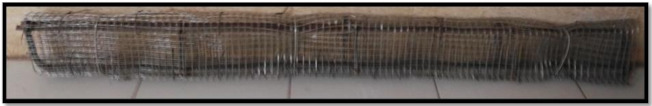
C3-A	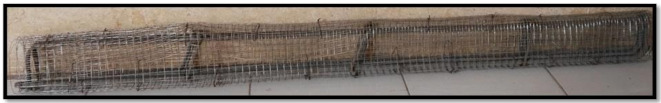
C4-B	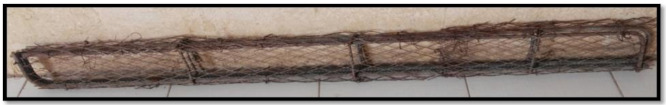
C5-B	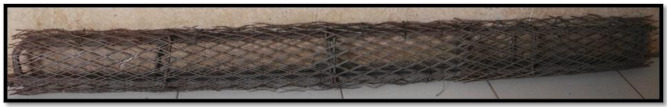
C6-C	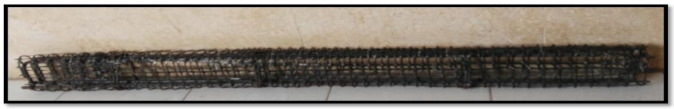
C7-D	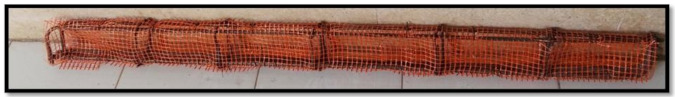
C8-D	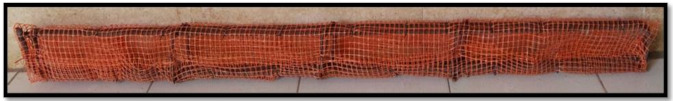

**Table 7:** Experimental test results.

Column	First Crack Load	Serviceability Load	Ultimate Load	Def. at First Crack Load	Def. at Ult. load	Ductility Ratio	Energy Absorption (kN·mm)
ID	(kN)	(kN)	(kN)	(mm)	(mm)	---	
C1	296.00	460.59	738.70	3.80	12.02	3.16	6320.08
C1-A	325.00	481.27	771.80	4.30	12.72	2.96	9201.48
C2-A	380.00	525.46	842.50	4.70	14.36	3.06	9353.37
C3-A	455.00	596.15	955.60	5.20	14.50	2.79	10,380.91
C4-B	443.00	507.46	813.70	5.90	15.45	2.62	10,122.72
C5-B	449.00	574.96	921.70	6.30	16.96	2.69	12,008.52
C6-C	515.00	640.90	1027.20	4.40	13.55	3.08	10,414.93
C7-D	391.00	496.65	796.40	3.90	12.08	3.10	6983.16
C8-D	378.00	524.71	841.30	4.50	14.07	3.13	9179.36

**Table 8:** Analytical results.

Column	First Crack Load	Ultimate Load	Def. at First Crack Load	Def. at Ult. Load	Ductility Ratio	Energy Absorption (kN·mm)
ID	(kN)	(kN)	(mm)	(mm)	---	
C1	260.30	812.57	2.85	8.12	2.85	4104.8310
C1-A	260.30	848.98	3.23	10.30	3.19	6200.2411
C2-A	260.30	906.95	3.53	10.50	2.98	7055.3147
C3-A	260.30	1051.16	3.90	10.31	2.64	7303.0707
C4-B	260.30	995.07	4.43	10.49	2.37	6899.9601
C5-B	260.30	1013.87	4.73	9.63	2.04	6396.8092
C6-C	260.30	1129.90	3.30	7.30	2.21	4933.7318
C7-D	260.30	876.04	2.93	7.76	2.65	4237.6949
C8-D	260.30	925.43	3.38	6.82	2.02	3782.9131

**Table 9:** Experimental and analytical results.

Column	First Crack Load	First Crack Load	Ultimate Load	Ultimate Load	Def. at Ult. Load	Def. at Ult. Load
ID	(kN)	(kN)	(kN)	(kN)	(mm)	(mm)
	NLA.	EXP.	NLA.	EXP.	NLA.	EXP.
C1	260.30	296.00	812.57	738.70	8.12	12.02
C1-A	260.30	325.00	848.98	771.80	10.30	12.72
C2-A	260.30	380.00	906.95	842.50	10.50	14.36
C3-A	260.30	455.00	1051.16	955.60	10.31	14.50
C4-B	260.30	443.00	995.07	813.70	10.49	15.45
C5-B	260.30	449.00	1013.87	921.70	9.63	16.96
C6-C	260.30	515.00	1129.90	1027.20	7.30	13.55
C7-D	260.30	391.00	876.04	796.40	7.76	12.08
C8-D	260.30	378.00	925.43	841.30	6.82	14.07
